# Emergence of Enteroaggregative Escherichia coli within the ST131 Lineage as a Cause of Extraintestinal Infections

**DOI:** 10.1128/mBio.00353-20

**Published:** 2020-05-19

**Authors:** Erik J. Boll, Søren Overballe-Petersen, Henrik Hasman, Louise Roer, Kim Ng, Flemming Scheutz, Anette M. Hammerum, Arnold Dungu, Frank Hansen, Thor B. Johannesen, Abigail Johnson, Divek T. Nair, Berit Lilje, Dennis S. Hansen, Karen A. Krogfelt, Timothy J. Johnson, Lance B. Price, James R. Johnson, Carsten Struve, Bente Olesen, Marc Stegger

**Affiliations:** a Department of Bacteria, Parasites and Fungi, Statens Serum Institut, Copenhagen, Denmark; b Department of Clinical Microbiology, Herlev and Gentofte Hospital, University of Copenhagen, Copenhagen, Denmark; c Department of Veterinary and Biomedical Sciences, University of Minnesota, Saint Paul, Minnesota, USA; d Department of Animal Sciences, University of Minnesota, Saint Paul, Minnesota, USA; e Division of Pathogen Genomics, Translational Genomics Research Institute, Flagstaff, Arizona, USA; f Department of Environmental and Occupational Health, Milken Institute School of Public Health, The George Washington University, Washington, DC, USA; g VA Medical Center, Minneapolis, Minnesota, USA; h University of Minnesota, Minneapolis, Minnesota, USA; Nanyang Technological University

**Keywords:** *E. coli*, ESBL, genomic, H27, resistance, ST131, enteroaggregative, evolution, outbreak, plasmids

## Abstract

Escherichia coli sequence type 131 (ST131) is a major cause of urinary and bloodstream infections. Its association with extended-spectrum β-lactamases (ESBLs) significantly complicates treatment. Its best-described component is the rapidly expanding *H*30Rx clade, containing allele 30 of the type 1 fimbrial adhesin gene *fimH*. This lineage appears to have emerged in the United States and spread around the world in part due to the acquisition of the ESBL-encoding *bla*_CTX-M-15_ gene and resistance to fluoroquinolones. However, non-*H*30 ST131 sublineages with other acquired CTX-M-type resistance genes are also emerging. Based on whole-genome analyses, we describe here the presence of an (*fimH*) *H*27 E. coli ST131 sublineage that has recently caused an outbreak of community-acquired bacteremia and recurrent urinary tract infections (UTIs) in Denmark. This sublineage has acquired both a virulence plasmid (pAA) that defines the enteroaggregative E. coli (EAEC) diarrheagenic pathotype and multiple genes associated with extraintestinal E. coli (ExPEC); combined, these traits have made this particular ST131 sublineage successful at colonizing its human host and causing recurrent UTI. Moreover, using a historic World Health Organization (WHO) E. coli collection and publicly available genome sequences, we identified a global *H*27 EAEC ST131 sublineage that dates back as far as 1998. Most *H*27 EAEC ST131 isolates harbor pAA or pAA-like plasmids, and our analysis strongly implies a single ancestral acquisition among these isolates. These findings illustrate both the profound plasticity of this important pathogenic E. coli ST131 *H*27 sublineage and genetic acquisitions of EAEC-specific virulence traits that likely confer an enhanced ability to cause intestinal colonization.

## Abstract (precis)

E. coli ST131 is an important extraintestinal pathogenic lineage. A signature characteristic of ST131 is its ability to asymptomatically colonize the gastrointestinal tract and then opportunistically cause extraintestinal infections, such as cystitis, pyelonephritis, and urosepsis. In this study, we identified an ST131 *H*27 sublineage that has acquired the enteroaggregative diarrheagenic phenotype, spread across multiple continents, and caused multiple outbreaks of community-acquired ESBL-associated bloodstream infections in Denmark. The strain’s ability to both cause diarrhea and innocuously colonize the human gastrointestinal tract may facilitate its dissemination and establishment in the community.

## INTRODUCTION

Escherichia coli sequence type 131 (ST131), which is the dominant multidrug-resistant (MDR) extraintestinal pathogenic E. coli (ExPEC) lineage worldwide, causes a wide range of infections, including bloodstream and urinary tract infections (BSIs/UTIs) (1–3). Its rise to global dominance and pathogenicity presumably was primed by the sequential acquisition of virulence-associated genes and then antibiotic resistance (4, 5).

ST131 exhibits predominantly serotype O25:H4 and is closely associated with fluoroquinolone resistance and the CTX-M-15 extended-spectrum β-lactamase (ESBL) (2, 6). The global expansion of E. coli ST131 has been driven mainly by a single clade, designated *H*30 because of its tight association with allele 30 of the type 1 fimbrial adhesin gene, *fimH*. *H*30, also described as clade C, has a prominent MDR clade, *H*30R, which accounts for most fluoroquinolone resistance within ST131. *H*30R, in turn, has two main subclades: *H*30R, of which most isolates are ESBL negative, although some produce CTX-M-14 or CTX-M-27, and *H*30Rx, which accounts for almost all ST131-associated CTX-M-15 production (6). In addition to *H*30, other distinct clades of ST131 are also circulating worldwide, most commonly associated with *fimH* alleles 41 and 22, also previously designated clades A and B, respectively (7). While these non-*H*30 clades are usually not associated with carriage of CTX-M genes, ST131-*H*22 and -*H*41 strains carrying *bla*_CTX-M-14_, *bla*_CTX-M-15_, or *bla*_CTX-M-27_ have been reported (4, 8), as have been food-associated ST131-*H*22 strains that carry *bla*_TEM-1_ (9).

ST131 isolates typically carry multiple ExPEC-associated virulence genes encoding adhesins, toxins, and siderophores but reportedly rarely carry virulence genes typical of diarrheagenic E. coli (DEC) (10, 11). However, among the 128 archival ST131 O25 isolates within an international World Health Organization (WHO) collection that we surveyed for temporal trends in antibiotic resistance and virulence traits, we identified 12 isolates (9%) that fulfilled molecular criteria for the enteroaggregative E. coli (EAEC) pathotype (12). Pulsed-field gel electrophoresis (PFGE) analysis revealed a cluster (∼80% similarity coefficient) comprising seven of these EAEC isolates. Of these seven isolates, six, including two urine isolates from patients with UTI, three fecal isolates from patients with diarrhea, and one lower respiratory tract isolate (associated symptoms unknown), were from Danish patients (1998 to 2000), supporting the occurrence in Denmark during these 3 years of an unrecognized EAEC ST131-associated outbreak of UTI and possibly also diarrhea (12).

EAEC strains are a well-established cause of endemic diarrheal illness in developing countries and of foodborne outbreaks in developed countries and more recently have been associated with extraintestinal infections (13). This pathotype gained particular attention following a major foodborne outbreak in Germany in 2011 that was caused by a Shiga toxin (Stx)-producing O104:H4 EAEC strain and resulted in 3,842 confirmed cases and 54 deaths (14). Additionally, it is concerning that a high prevalence of multidrug resistance among EAEC strains has been reported, and reports of CTX-M-type, ESBL-producing EAEC have emerged around the world (15–18).

EAEC pathogenesis involves adherence to human intestinal mucosa by virtue of aggregative adherence fimbriae (AAF) and subsequent biofilm formation. The AAF are located on a large plasmid, designated pAA, that also encodes a suite of other EAEC virulence factors. These include AggR, a global regulator of EAEC virulence; dispersin, required for proper dispersal of AAFs on the bacterial surface; the AatPABCD transporter system, which mediates dispersin secretion; and Aar, a recently described negative regulator of AggR (19, 20).

To understand the emergence and underlying genetic acquisitions leading to ESBL-producing EAEC ST131, we investigated the relatedness of a large collection of temporally and spatially diverse ST131 isolates. This investigation revealed the emergence of a global *H*27 sublineage of EAEC linked to a single acquisition of a pAA plasmid that likely improved the strain’s ability to persistently colonize its human host.

## RESULTS

### Identification of historic Danish EAEC ST131 isolates.

Our previous PFGE-based analysis of ST131 O25 isolates within the WHO E. coli collection identified an apparently unrecognized outbreak of UTI, and possibly also diarrhea, in Denmark in 1998 to 2000 that was caused by a group of seemingly similar EAEC isolates (12). To better estimate these isolates’ relatedness and to correct for recombination (6), all 128 ST131 isolates from the WHO collection were subjected to whole-genome sequencing. *In silico* analysis of the genome sequences revealed that the most frequent *fimH* alleles were *H*30 (51%, *n* = 65), *H*22 (34%, *n* = 44), and *H*27 (7%, *n* = 9). Most isolates harbored multiple antibiotic resistance genes. The most common ESBL gene was *bla*_CTX-M-15_ (37%, *n* = 47), which occurred almost exclusively within the *H*30 subgroup (98%, 46/47) (see Table S1 in the supplemental material).

10.1128/mBio.00353-20.6 TABLE S1 Metadata for all E. coli ST131 isolates (*n* = 128) from the WHO E. coli and *Klebsiella* Centre’s E. coli collection (1968 to 2011). Download Table S1, XLS file, 0.1 MB. Copyright © 2020 Boll et al. 2020 Boll et al. This content is distributed under the terms of the Creative Commons Attribution 4.0 International license.

To ensure that only high-quality genome data sets were included in our single-nucleotide polymorphism (SNP) analysis, we excluded the genome data for 7 (5%) of the 128 WHO ST131 isolates due to low sequencing depth. To supplement the remaining 121 WHO isolate genome sequences, we included 93 genome sequences from a published collection of human clinical ST131 E. coli isolates from the United States and Germany (6), giving 214 genomes in total. Across these genomes, 11,529 SNPs were identified within ∼38% of the ST131 *H*30Rx JJ1886 reference chromosome that was found to be conserved among the study genomes after applying quality parameters during SNP calling. The identification and purging of recombinant regions left 4,241 phylogenetically informative SNPs, which were used to infer relationships between the isolates.

**FIG 1:**
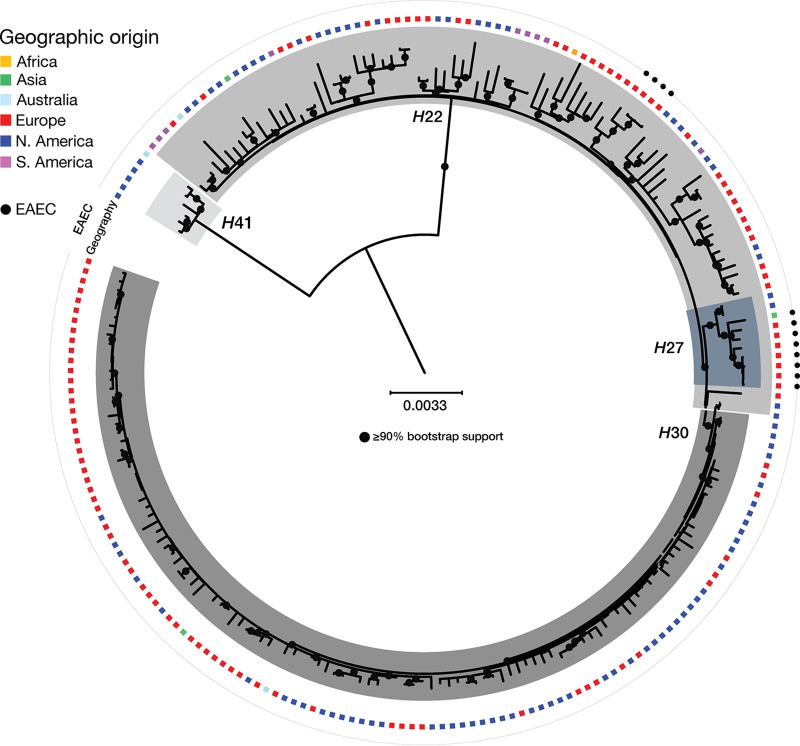
Phylogeny of international Escherichia coli ST131 strains. Rooted phylogenetic tree of ST131 E. coli strains from the WHO collection (*n* = 121) and the U.S./German collection (*n* = 93) based on 11,529 SNPs. Geographical origins are shown as colored squares, and isolates fulfilling the molecular criteria for EAEC are marked with a black circle. The scale bar represents substitutions per site.

The SNP-based phylogeny showed distinct overall clustering of isolates in accordance with their *fimH* alleles. All 116 isolates carrying *fimH*30 clustered as a monophyletic clade, which we designated *H*30 (Fig. 1). Likewise, all 10 isolates carrying *fimH*27 clustered within a monophyletic clade, which we designated *H*27, that, like the *H*30 clade, appeared to descend from an ancestral *H*22 group. Of these *H*27 isolates, eight, all from the WHO collection, qualified molecularly as EAEC based on the presence of  ≥1 of the EAEC-associated putative virulence genes *aggR*, *aatA*, and *aaiC* (21) and formed a separate subclade (within the *H*27 clade) from the two non-EAEC *H*27 strains. This *H*27 EAEC cluster comprised a fecal isolate from Vietnam (C86-04 from 2004) and seven Danish isolates, including two urine isolates, one lower-respiratory-tract isolate, and four fecal isolates from patients with diarrhea. This range of clinical sources suggested that this sublineage was able to cause both extraintestinal infections and diarrhea.

The SNP-based analysis also identified an additional monophyletic EAEC cluster located within the *H*22 sublineage that consisted of the remaining four EAEC isolates from the WHO collection (Fig. 1). Three of the isolates carried *fimH*22, whereas one isolate carried *fimH*298, which differs from *fimH*22 by only a single nucleotide. All four isolates were from urine samples collected within a 10-month period in 1998 from elderly patients admitted to four different departments of the same hospital in the Capital Region of Denmark, suggesting an unrecognized nosocomial UTI outbreak. With the exception of the single Vietnamese isolate, all 12 EAEC isolates were recovered from Danish patients. The Vietnamese isolate carried *bla*_CTX-M-27_, whereas the 11 Danish EAEC isolates carried no ESBL genes (Table S1).

### EAEC virulence genes in historic EAEC ST131 isolates.

Among the 12 ST131 EAEC isolates, the four clustered EAEC urine isolates that carried *fimH*22 or *fimH*298 all harbored the *aggDCBA* gene cluster encoding the AAF/I variant, whereas the eight *H*27 EAEC isolates of diverse sources all harbored *agg5DCBA*, encoding the recently described AAF type V variant (AAF/V) (22) (Table S2). Likewise, all but two contained genes encoding AggR (*aggR*), Aar, dispersin (*aap*), and the AatPABCD transporter system (*aat* gene cluster), plus two additional AggR-regulated open reading frames (ORFs), ORF3 and ORF4, that are assumed to play a role in isoprenoid biosynthesis (23). The two exceptional isolates, C1883-99 (*H*27) and C167-00 (*H*22), lacked most of these EAEC-specific genes.

10.1128/mBio.00353-20.7 TABLE S2 Key genetic profile data for EAEC ST131 isolates. Download Table S2, XLSX file, 0.02 MB. Copyright © 2020 Boll et al. 2020 Boll et al. This content is distributed under the terms of the Creative Commons Attribution 4.0 International license.

### Invasive *bla*_CTX-M-101_-containing EAEC ST131 isolates from Danish patients.

To determine whether EAEC ST131 strains were present among contemporary Danish patients, we searched for ST131- and EAEC-specific virulence genes within a published collection of 552 whole-genome-sequenced ESBL-producing E. coli isolates from Danish patients with bloodstream infections (BSIs) between 2014 and 2015 (24). ST131 accounted for 50% of the isolates (24). Among the 258 ST131 isolates, 25 (9.7%) qualified molecularly as EAEC, of which 24 (9%) harbored the genes encoding AAF/V, AggR, Aar, Aap, AatPABCD, and ORF3/4. The remaining isolate contained the genes encoding AatPABCD (Table S2). All 25 isolates carried *fimH*27 and *bla*_CTX-M-101_. In total, 27 isolates in the collection were *bla*_CTX-M-101_ positive, of which 93% qualified molecularly as EAEC. The EAEC pathotype did not occur in conjunction with any other ESBL-encoding gene. As shown by Roer et al., the 27 ST131 strains with *bla*_CTX-M-101_ formed a distinct cluster with nine or fewer SNP differences (24), supporting a recent emergence or clonal spread.

To assess whether a corresponding CTX-M-101-producing EAEC ST131 strain could be detected in the urine of the source patients for the blood isolates, genome sequencing was performed on all available urine isolates from 8 of the 25 patients with a CTX-M-101-producing EAEC ST131 BSI. Notably, as all eight patients had presented with UTI on one or more occasions, multiple ST131-positive urine samples were available for four of the patients. The urine samples were taken in a time frame ranging from 3 weeks prior to 8 months after the BSI episode. SNP analysis demonstrated that pairs of blood and urine isolates from the same patient were nearly identical (i.e., differed by ≤6 SNPs), whereas different pairs were separated by 0 to 11 SNPs. The respective blood and urine isolates from five of the eight cases were nearest neighbors in the phylogeny, strongly implying their distinct relatedness (Fig. S1). For the remaining three cases, the clustering was inconclusive. Combined, these findings suggest strongly that the ESBL-producing EAEC ST131 strains were capable of long-term persistence in these hosts and/or their immediate environment and of causing repeated episodes of UTI.

10.1128/mBio.00353-20.1 FIG S1 Relatedness of blood and urine ST131 EAEC *H*27 isolates. Midpoint-rooted phylogeny based on 58 SNPs of paired blood (red) and urine (yellow) isolates from eight Danish cases as well as all remaining Danish ST131 EAEC *H*27 *bla*_CTX-M-101_-positive isolates. Black dots do not represent samples but are inserted for visual clarity. The analysis depicts the distinct relatedness of five corresponding blood and urine isolates (cases 1, 4, 5, 7, and 8), whereas the data are inconclusive for the remaining isolates (cases 2, 3, and 6). Sampling dates for urine samples are presented relative to date of blood sampling. Download FIG S1, PDF file, 0.4 MB. Copyright © 2020 Boll et al. 2020 Boll et al. This content is distributed under the terms of the Creative Commons Attribution 4.0 International license.

### pAA plasmid characterization in ST131 isolates.

To characterize in detail the pAA plasmids present in the *H*27 ESBL-producing EAEC ST131 strains, we applied MinION sequencing (Oxford Nanopore Technologies) to obtain the complete plasmid sequences for five *bla*_CTX-M-101_-containing EAEC isolates and five EAEC isolates from the WHO collection. The plasmid from a single representative *bla*_CTX-M-101_-containing isolate (ESBL20150001) was designated pAA-ST131 and used for in-depth *in silico* analyses.

**FIG 2:**
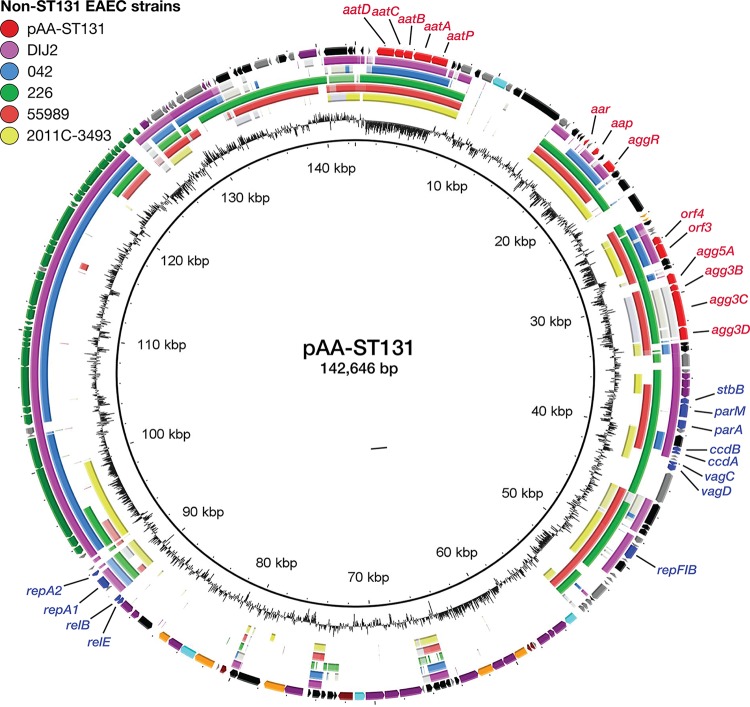
Comparison of pAA plasmid content. Circular map of plasmid pAA-ST131 compared to other publicly available pAA plasmids from non-ST131 EAEC type strains. The outer ring shows predicted ORFs of pAA-ST131. Colors represent different putative functions: gray, hypothetical proteins; red, EAEC-specific virulence factors; blue, plasmid replication and maintenance; maroon, catabolism and metabolism; orange, membrane and transporter proteins; green, conjugational transfer proteins (*tra* and *trb* genes); light blue, regulatory genes; purple, miscellaneous; and black, mobile elements. Within the circles representing pAA plasmids from other EAEC strains, the darkest color indicates >90% nucleotide identity, the lightest color >80% identity.

The complete sequence of pAA-ST131 was 142,646 bp in length, with an average G+C content of 48.7% (GenBank accession no. KY706108) (Fig. 2). A total of 197 ORFs were predicted and annotated, and 142 were assigned functionally. Two replicons were identified, RepFII and RepFIB, with the multireplicon F plasmid FAB pMLST formula of F1:A-:B33. The pAA-ST131 plasmid contained several genes associated with plasmid stability, including *parA*, *parM*, and *stbB*, plus three toxin-antitoxin (TA)-based addiction systems: *ccdAB*, *vagCD*, and *relBE* (Fig. 2). Furthermore, pAA-ST131 harbored a 33-kb complete *tra* region encoding transfer components (24 *tra* genes, 8 *trb* genes, and *finO*), implying that the plasmid is transmissible. It also contained genes encoding putative transmembrane proteins and proteins involved in catabolism and metabolism, plus several integrated mobile elements. It contained, as expected, all of the EAEC virulence factor genes already identified by Illumina sequencing but no additional known virulence factor genes or antibiotic resistance genes, including *bla*_CTX-M-101_.

A BLASTP comparison was made of pAA-ST131 with five pAA plasmids from EAEC-type strains (each containing *aggR*, *aap*, and genes encoding an AAF variant), i.e., GenBank accession numbers (strain name) NC_018666 (strain 2011C-3493), NC_011752 (strain 55989), FN554767 (strain 042), and NC_008460 (strain DIJ1) and Sequence Read Archive (SRA) number SRA055981 (strain 226). This analysis showed that all these plasmids shared common features (Fig. 2), including EAEC virulence genes, but also varied substantially in genetic content, as described previously for E. coli virulence plasmids (25). Interestingly, regions containing putative metabolic, catabolic, and transmembrane proteins were unique to pAA-ST131. Although the sequences of the associated chromosomes were available for typing for only some of these EAEC isolates, analysis of available data showed that the isolates represented at least three unrelated STs (ST40, ST414, and ST678). Additionally, although, like pAA-ST131, four of the five reference pAA plasmids also contained the RepFIB and/or RepFIIA replicon, only three contained both, and only two contained the *tra* region.

Standard agarose gel-based plasmid profiling demonstrated that all 10 of the ST131 MinION-sequenced WHO- or *bla*_CTX-M-101_-containing EAEC strains, for which complete plasmid sequences were obtained, contained only a single plasmid of various sizes (Fig. S2). Isolates ESBL20150196 and ESBL20150300 (both obtained from Danish patients in 2015) each harbored a single 31-kb plasmid, which, based on *in silico* mapping, was the result of a single major deletion that resulted in the loss of all EAEC-specific virulence genes (Fig. S3). The two remaining ESBL-producing isolates, plus three of the WHO collection isolates, harbored plasmids nearly identical to pAA-ST131 (ESBL20150001). Isolates C546-00 and C1883-99, from the WHO collection, had slightly truncated plasmids in which a single or double deletion event, respectively, had resulted in the loss of some EAEC-specific genes (Fig. S3). None of the ESBL-producing isolates contained additional plasmids (Fig. S2), implying a chromosomal localization for *bla*_CTX-M-101_.

10.1128/mBio.00353-20.2 FIG S2 Gel electrophoresis-based profiling of plasmids from representative *fimH*27-carrying ST131 isolates. Five isolates containing *bla*_CTX-M-101_ and five isolates from the WHO collection were included. A plasmid profile with Escherichia coli 39R861 as a marker (147, 63, 36, and 7kb) is included in lane 1. All 10 *fimH*27 strains harbor only a single plasmid. In seven of the strains, this plasmid size corresponds to 140 to 145 kb in size, whereas in WHO isolate C1883-99 it is slightly smaller. Isolates ESBL20150196 and ESBL20150300 both harbored a substantially smaller plasmid of approximately 30 kb. Download FIG S2, PDF file, 1.0 MB. Copyright © 2020 Boll et al. 2020 Boll et al. This content is distributed under the terms of the Creative Commons Attribution 4.0 International license.

10.1128/mBio.00353-20.3 FIG S3 Alignment of pAA-ST131 plasmids. Schematic representation of closed plasmids from representative *fimH*27-carrying ST131 isolates containing *bla*_CTX-M-101_ or from the WHO collection (same strains as those shown in Fig. S2). The locations of EAEC-specific virulence genes on pAA-ST131 are highlighted, as are plasmid insertions. Download FIG S3, PDF file, 0.4 MB. Copyright © 2020 Boll et al. 2020 Boll et al. This content is distributed under the terms of the Creative Commons Attribution 4.0 International license.

### Global emergence of EAEC ST131 isolates carrying *fimH*27.

To assess the global extent of EAEC ST131 isolates, we screened for *aggR* (the global regulator of EAEC virulence) among all >3,500 E. coli ST131 genomes (as of 15 November 2017) available in EnteroBase (http://enterobase.warwick.ac.uk), a database with ∼80,0000 genomic assemblies of E. coli. Disregarding the *aggR*-positive, *bla*_CTX-M-101_-containing Danish EAEC isolates already present in the database, we identified another 25 international (i.e., non-Danish) *aggR*-positive ST131 isolates, which carried the following *fimH* alleles (no. of isolates, percentage of 25 isolates): *fimH*27 (18, 66%), *fimH*22 (1, 4%), *fimH*298 (2, 8%), *fimH*30 (1, 4%), *fim*H5 (2, 8%), and *fim*H54-like (1, 4%) (Table S2).

**FIG 3:**
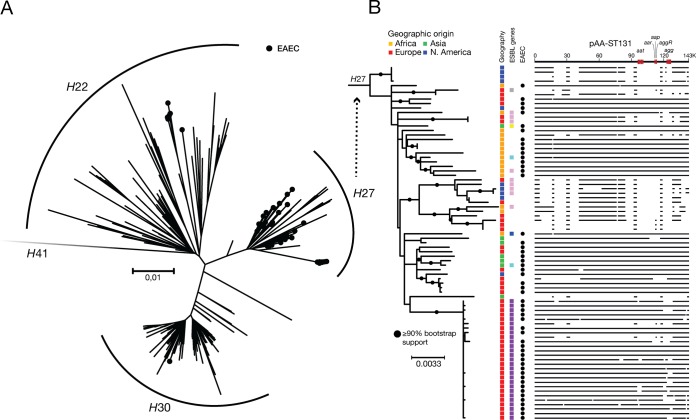
Genetic context and features of Escherichia coli ST131 *H*27. (A) Unrooted phylogenetic tree of 287 ST131 genomes. Isolates are from the WHO collection, the U.S./German collection, the Danish *bla*_CTX-M-101_-containing isolate set from 2014 to 2015, all international *fimH*27 isolates and all non-*H*27, and *aggR*-positive ST131 isolates from EnteroBase. The tree is based on 4,381 SNPs after purging for recombination. The distant *H*41 cluster is not shown. EAEC isolates are highlighted in black. (B) Rooted phylogenetic tree with all isolates within the *H*27 clade (*n* = 79) based on 842 SNPs after purging for recombination. ESBL genes include CARB-2 (gray), CTX-M-101 (purple), CTX-M15 (pink), CTX-M-15+OXA-10 (dark blue), CTX-M-27 (yellow), and SHV-12 (light blue). Geographical origins and isolates fulfilling the molecular criteria for EAEC are highlighted, as are the results of sequence read mapping against the pAA-ST131 reference plasmid. The scale bar represents substitutions per site.

We next assessed whether the 12 EAEC ST131 isolates from the WHO collection were clonally related to other available EAEC ST131 isolates. For this, a SNP-based phylogeny was constructed, including (i) all 121 ST131 isolates from the WHO collection (12); (ii) the 93 German/U.S. ST131 isolates (6); (iii) the 27 Danish *bla*_CTX-M-101_-containing ST131 isolates from 2014 to 2015 (24); (iv) all 42 international *fimH*27-carrying ST131 isolates from EnteroBase; and (v) all seven non-*H*27, *aggR*-positive ST131 isolates from EnteroBase. Focusing on isolates within the *H*22 clade (Fig. 3A), WHO collection EAEC isolate C180-00, carrying *fimH*298, clustered together with two *fimH*298-carrying EnteroBase EAEC isolates (SRA numbers SRR2970775 and SRR2970774) from Cambodia (2009 to 2010). In contrast, the three *fimH22*-carrying WHO collection EAEC isolates were not closely related to the only *fimH22*-carrying EnteroBase EAEC isolate.

In the unrooted phylogeny, most EAEC isolates nested within the *H*27 clade (Fig. 3A). To improve resolution, this clade’s 79 isolates (76 with *fimH*27, 2 with *fimH*5, 1 with *fimH*54) were analyzed separately. The 27 *bla*_CTX-M-101_-containing Danish EAEC isolates formed a distinct cluster (Fig. 3B). In contrast, none of the *fimH*27-carrying international isolates clustered either with the Danish isolates or with one another, suggesting that the outbreak was confined to Denmark and that no other focal outbreaks were captured. Instead, the WHO collection *H*27 EAEC isolates (11 Danish and 1 Vietnamese) were intermingled with isolates from the United Kingdom, Thailand, and Canada, suggesting the global spread of a common-ancestry strain.

To determine the mosaicism of the EAEC-specific virulence genes among *fimH*27-carrying ST131 isolates, sequence reads from all 79 *H*27 clade isolates were mapped against pAA-ST131 from ESBL20150001. Intriguingly, genes of pAA-ST131 were highly conserved across the *H*27 EAEC isolates (Fig. 3B) when assessed based on continuous reference mappings of >1,000 bp in length using the Northern Arizona SNP Pipeline (NASP) against the pAA-ST131 reference plasmid. We next performed a SNP-based analysis of pAA-ST131 across all 57 *H*27 isolates with more than 70% coverage of the plasmid, with three *H*22 clade EAEC isolates from the WHO collection used as an outgroup. This identified 197 SNPs within the ∼25% of pAA-ST131 that were conserved across all isolates, equivalent to ∼1 SNP per 4,000 bp conserved plasmids among the *H*27 isolates compared to 1 per ∼5,000 in the core chromosome of the same isolate collection. Phylogenetic analysis based on these SNPs showed that pAA-ST131 is highly conserved among the *H*27 isolates but differs considerably between the *H*27 and non-*H*27 ST131 isolates (Fig. S4), strongly suggesting a single acquisition of the plasmid by an *H*27 clade ancestor, with subsequent partial deletions within certain descendants.

10.1128/mBio.00353-20.4 FIG S4 pAA diversity among ST131 *H*27 isolates. Phylogenetic tree of pAA from ST131 *H*27 isolates with more than 70% coverage of pAA-ST131 from representative Danish isolate ESBL20150001. Three of the *H*22/*H*298 EAEC isolates from the WHO collection (C156-00, C168-00, and C180-00) were included as an outgroup. A total of 197 SNPs were identified in approximately 25% of pAA-ST131, which was conserved across all isolates after application of quality measurements for inclusion. Download FIG S4, PDF file, 0.7 MB. Copyright © 2020 Boll et al. 2020 Boll et al. This content is distributed under the terms of the Creative Commons Attribution 4.0 International license.

Finally, we performed a BLASTN analysis to screen the *H*27 isolates for classical ExPEC virulence genes (Table S3). All *H*27 isolates carried *sfa*, *iutA*, and *kpsM* II, which qualified them molecularly as ExPEC (26). In addition, they all contained *chuA*, *fyuA*, and *yfcV*, which, moreover, qualified them molecularly as uropathogenic E. coli (UPEC) (27).

10.1128/mBio.00353-20.8 TABLE S3 ExPEC virulence genes present in the ST131 *fimH*27 isolates of Fig. 2B. Download Table S3, XLSX file, 0.01 MB. Copyright © 2020 Boll et al. 2020 Boll et al. This content is distributed under the terms of the Creative Commons Attribution 4.0 International license.

### Phenotypic confirmation of pAA plasmid-mediated adhesive properties.

To determine whether the AAF encoded by the pAA plasmids in *H*27 isolates is functional and potentially facilitates adherence to the intestinal mucosa, we compared the ability of two of the ESBL EAEC ST131 isolates, ESBL20150001 and ESBL20150196, carrying a full-length and truncated pAA plasmid lacking the AAF-encoding genes, respectively, to adhere to T84 colonic epithelial cells. ESBL20150001 adhered significantly better than did ESBL20150196, whereas both isolates adhered significantly better than did a laboratory E. coli K-12 strain (Fig. S5).

10.1128/mBio.00353-20.5 FIG S5 Adhesion of ESBL-producing EAEC ST131 isolates to T84 colonic epithelial cells. ESBL2015001 harbors the full-length pAA-ST131 plasmid, whereas ESBL20150196 harbors a truncated plasmid that lacks all EAEC-specific virulence genes. A laboratory E. coli K-12 strain was included as a reference strain. *, *P ≤ *0.05; **, *P ≤ *0.01. Download FIG S5, PDF file, 0.6 MB. Copyright © 2020 Boll et al. 2020 Boll et al. This content is distributed under the terms of the Creative Commons Attribution 4.0 International license.

## DISCUSSION

E. coli ST131 is an extensively reported MDR ExPEC lineage associated with both UTI and BSI (1). Many typical ExPEC-associated virulence factors, including P fimbriae, hemolysins, and factors conferring increased serum survival and iron uptake, have been identified in ST131 isolates (11, 12), but overall little is known about which genes (whether they promote virulence or other phenotypes) have made this clonal lineage so successful. In contrast, virulence traits associated with diarrheagenic E. coli (DEC) pathotypes have thus far largely been absent from ST131 isolates (12). Their absence might be considered surprising because ST131 sublineages vary extensively according to acquired virulence genes (2, 28) and exhibit high levels of genomic plasticity. This plasticity includes frequent recombination and plasmid flux, particularly involving IncF-type plasmids, which facilitates the spread of antibiotic resistance and virulence genes (4, 29). Importantly, in ST131 the fitness costs that result from these genomic events are offset by compensatory mutations (7). Here, we document the ST131 *H*27 sublineage to have acquired the enteroaggregative diarrheagenic phenotype followed by spread across multiple continents and to have caused outbreaks of community-acquired ESBL-associated bloodstream infections in Denmark.

In developed countries, EAEC is known mostly as a cause of mild to moderately severe, self-limiting diarrhea. Indeed, long-term carriage of EAEC has been suggested to lead to colonization rather than infection (13). In contrast, in developing countries EAEC is a leading cause of childhood diarrhea (13, 30). The pathogenic potential of EAEC is underscored by its ability to cause major foodborne outbreaks of diarrheal disease (21, 31, 32). Intriguingly, recent studies have associated EAEC with UTI, suggesting that what classically has been regarded as a DEC pathotype also qualifies as ExPEC and causes both diarrhea and UTI (33–35).

Like other DEC pathotypes, EAEC has been shown to encompass diverse genetic lineages, reflecting a high level of phylogenetic heterogeneity (18, 36). Although until recently no EAEC ST131 associated with extraintestinal infections had been documented, recent reports have described the occurrence in certain ST131 sublineages with EAEC-specific traits in geographically distinct areas (12, 15, 18). ESBL-producing ST38 strains, and other sequence types of various serotypes, have been implicated as emerging hybrids of UPEC and EAEC that cause mainly UTI in Germany, the Netherlands, and the United Kingdom (37). In a study of fecal and urine isolates from Danish patients in 1998 to 2000, we made the novel observation of EAEC ST131 isolates of serotype O25 (12). Subsequently, another study documented a *bla*_CTX-M-14_-containing EAEC ST131 O25:H4 strain in stool samples of diarrheic patients in Japan from 2003 onwards (15).

Our previous PFGE analysis of Danish ST131 O25 E. coli isolates from the WHO collection demonstrated that 7 of the 12 identified EAEC strains were highly similar and appeared to have been part of an unrecognized UTI and diarrhea outbreak in Denmark from 1998 to 2000 (12). Although the genomic approach we used here to analyze the same isolates confirmed the suspected outbreak, it also yielded certain substantially different conclusions than those from the PFGE analysis, including the identification of a second distinct cluster of EAEC urine isolates. These discrepancies between WGS SNP-based analysis and PFGE analysis among ST131 isolates correspond with previous findings (6).

Here, we also document the current presence of an *H*27 ESBL-producing EAEC ST131 sublineage as a cause of bacteremia in patients admitted to hospitals in all five regions of Denmark. The isolates, collected across Denmark over a 16-month period in 2014 to 2015, all carried *bla*_CTX-M-101_. Notably, heretofore the only other reports of CTX-M-101-positive E. coli were from China (38–40). Unfortunately, travel information was not available for all the Danish patients with CTX-M-101-producing E. coli. Roer et al. used a genomic approach to establish that the Danish ESBL EAEC isolates were highly clonal, strongly suggesting a recent common source (24). Here, by analyzing sequential urine isolates from eight of the Danish patients with ESBL-producing EAEC ST131 *H*27 bacteremia, we found that five patients had recurrent UTI episodes caused by their BSI strain that were separated in time by up to 8 months from one another and/or the BSI episode. The most plausible explanation for this phenomenon is that the Danish ESBL-producing EAEC ST131 outbreak strain is capable of persistently colonizing patients, resulting in occasional clinical manifestations, although conceivably the patients may have been exposed repeatedly to an external source.

Using the EnteroBase collection of available ST131 genome sequences, we identified the global presence of *fimH*27-carrying EAEC ST131 isolates. These isolates originated from Africa, Asia, Europe, and North America, and, together with the Danish EAEC ST131 outbreak isolates, spanned more than 2 decades. Strikingly, all the EAEC ST131 *H*27 strains studied here proved to be clonal, i.e., to share an ancestor. Moreover, they share a virulence plasmid, which we designated pAA-ST131, which appears to have been acquired once in the *H*27 clade and transmitted vertically, although it exhibits considerable variation in some isolates. MinION-based sequencing of the pAA-ST131 plasmid in representative ESBL-producing EAEC ST131 strains identified an array of classical plasmid-encoded EAEC-defining virulence genes, including those encoding AAFs and the global regulator of virulence AggR. The AAFs play a central role in EAEC pathogenesis in part by facilitating adherence to the intestinal mucosa (20). Supporting a similar potential role in the EAEC ST131-*H*27 clade, a representative Danish ESBL-producing EAEC isolate harboring a full-length pAA-ST131 plasmid adhered significantly better to colonic epithelial cells than another closely related Danish outbreak strain harboring a truncated plasmid without the AAF-encoding genes.

The EAEC ST131 sublineage described here is reminiscent of an E. coli O78:H10 strain that caused a UTI outbreak in Copenhagen in 1991, in that both fulfilled the molecular criteria for EAEC and also contained multiple ExPEC virulence genes (34). That the O78:H10 outbreak strains’ EAEC-associated virulence factors increased uropathogenicity (35) suggests that this is true also for EAEC ST131 strains. The O78:H10 EAEC outbreak strain, however, was never found outside the Capital Region of Denmark and was never identified as having caused BSI. In contrast, ST131 has proved highly successful globally and is a leading cause of BSI, which makes the potential of future outbreaks of UTI and bacteremia caused by this *H*27 sublineage, or other cases of EAEC ST131, a cause for serious concern.

Despite the genetic evidence that all 25 *bla*_CTX-M-101_-containing Danish EAEC isolates were derived from a common ancestor, implying a common source, we have been unable to establish a patient link or explain the spread of the sublineage across regions using the limited available epidemiological data. From all but one of the 25 patients, an ESBL-producing E. coli was isolated from urine the same day that CTX-M-101-producing E. coli was detected from blood (data not shown). UTI is often caused by enteric E. coli strains that enter the urinary tract via the fecal-perineal-urethral route, and, in some instances, may have as their proximate external source food products or animals (41). The gender distribution among the present 25 cases (68% females) differs significantly (*P = *0.01 by Pearson’s χ^2^ test) from that across the entire collection of 258 ESBL-producing ST131 bloodstream isolates from Danish hospitals in 2014 to 2015 (42% females) (24). A similar overrepresentation of women was observed during the 2011 multinational European outbreak caused by a novel multipathotype, Shiga-toxin-producing EAEC O104:H4 strain (42), for which bean sprouts were the most likely vehicle of infection. Interestingly, the high proportion of female cases was thought to reflect the tendency of women to be more health-conscious, which, by analogy, suggests a food-related source for the Danish ESBL-producing EAEC ST31 outbreak. Further screening of, e.g., fecal and urine samples from patients who present with diarrhea or UTI, is warranted to determine the true clinical impact of ESBL-producing EAEC ST131-*H*27 strains and to provide the demographic and epidemiological data needed to identify potential sources.

In conclusion, we hypothesize that the acquisition of the pAA plasmid made the ESBL-producing EAEC ST131-*H*27 sublineage highly successful at persistently colonizing patients, thereby allowing it to occasionally cause UTIs and diarrhea. The presence of multiple ExPEC virulence factors, including P fimbriae, α-hemolysin, and Sat, in turn likely facilitates dissemination from the urinary tract to the bloodstream. Furthermore, we have demonstrated the ability of a non-*H*30 ST131 sublineage to acquire EAEC-specific pAA virulence plasmids and to disseminate across multiple continents over the past 2 decades. Apart from virulence/colonization-associated genes, this EAEC ST131-*H*27 strain has also acquired *bla*_CTX-M-101_ and has caused BSI outbreaks in several geographic regions in Denmark seemingly associated with recurrent infections. These findings emphasize the potential for pathogens to evolve, potentially generating important new pathotypes that require continuous vigilance.

## MATERIALS AND METHODS

### Bacterial isolates, sequencing, and genomic data.

WGS was performed on a previously described international collection of 128 ST131 E. coli human isolates of serotype O25:H4 or O25:H- (1968 to 2011) from the WHO Collaborating Centre for Reference and Research on *Escherichia* and *Klebsiella* (www.ssi.dk) (12). DNA samples were prepared for multiplexed, paired-end sequencing using a combination of Illumina MiSeq and HiSeq (6, 12). We also included sequence data from a published E. coli ST131 data set comprising 93 U.S. and German isolates of human origin (1967 to 2011) (6) and sequences from 27 ESBL-producing ST131 bloodstream isolates (carrying *bla*_CTX-M-101_) collected from Danish patients within the national surveillance program for antimicrobial resistance (DANMAP) for ESBL-producing E. coli (2014 to 2015) (24). Furthermore, data from all >3,500 E. coli ST131 genomes available at EnteroBase (http://enterobase.warwick.ac.uk, accessed 15 November 2017) were analyzed for EAEC characteristics (presence of the *aggR* gene) by BLASTN analysis, and positive isolates were included. Finally, we sequenced and included 13 E. coli isolates obtained from urine from the source patients for eight of the ESBL-producing blood isolates. The sequences were analyzed using the Bacterial Analysis Platform (BAP) from the Center for Genomic Epidemiology (43).

### Plasmid sequencing and analysis.

Plasmid DNA was sequenced on both a MiSeq instrument (Illumina) and a MinION flow cell (Oxford Nanopore Technologies). The MiSeq library was made using the Nextera XT kit (Illumina), and sequencing was performed as a paired-end 250-bp run, yielding 372,720 reads with an average length of 237 bp. The MinION library was prepared using the Genomic Sequencing SQK-MAP006 kit and was sequenced on a FLO-MAP003 early access flow cell according to the manufacturer’s instructions. Fast5 read files were subjected to base calling via a two-direction workflow using Metrichor software (ONT) to yield two-dimensional read files. Mixed assembly was performed by combining MiSeq and MinION reads using the SPAdes assembler (v3.9.0). Finally, CLCbio Genomics Workbench (v9.5.2) was used for end trimming of the assembled plasmid and for final error correction by mapping trimmed MiSeq reads against the plasmid contig obtained after the mixed SPAdes assembly.pAA-ST131 was annotated using RAST (44), with putative hypothetical genes curated manually using NCBI BLASTN and BLASTP searches. A BLASTN atlas of pAA-ST131 and other pAA virulence plasmids was constructed using BLAST Ring Image Generator v0.95 (BRIG) (45).

### Virulence genotyping.

Isolates qualified molecularly as EAEC if they were positive for ≥1 of the EAEC-associated putative virulence genes *aggR*, *aatA*, and *aaiC* (12). Isolates were regarded as ExPEC if they were positive for ≥2 of *papA* and/or *papC* (P fimbriae; counted as one), *sfa* and *foc* (S and F1C fimbriae; counted as one), *afa* and *dra* (Dr-binding adhesins; counted as one), *kspM* II (group 2 capsule), and *iutA* (aerobactin siderophore system) (26). Isolates were considered UPEC if positive for ≥2 of *chuA* (heme uptake), *fyuA* (yersiniabactin siderophore system), *vat* (vacuolating toxin), and *yfcV* (adhesin) (27).

### Identification of SNPs.

SNPs in the core chromosome or plasmid, depending on analysis, were identified using the Northern Arizona SNP Pipeline (NASP) (46). Briefly, duplicate regions of the reference chromosome of strain JJ1886 or pAA-ST131 plasmid (GenBank accession no. CP006784 and KY706108, respectively) were identified by aligning the reference against itself with NUCmer (47), followed by mapping of Illumina raw reads against the reference using the Burrows-Wheeler Aligner (BWA) (48) with identification of SNPs using GATK (49). Purging of recombinant regions in the chromosome-based SNPs was performed using Gubbins v2.2 (50).

### Phylogenetic analysis.

Relatedness between isolates according to core genome SNPs was inferred using RAxML v8.2.10 using the GTRCAT model (51) with 100 bootstrap replicates. Relatedness of the plasmids was inferred using PHyML with Smart Model Selection (52) with tree searching using subtree-pruning-regrafting and 100 bootstrap replicates. Plasmid sequences from all 10 MinION sequences were analyzed for deletion and insertion using the progressiveMauve algorithm in Mauve v1.4.0 as implemented in Geneious Prime v2020.0.4 (Biomatters, Ltd.).

### Plasmid profiling.

Plasmids were purified as described by Kado and Liu (53), visualized by separation on 0.8% agarose gel electrophoresis, and stained with GelRed (Biotium, Hayward, Ca, USA). E. coli strain 39R861, which contains four plasmids with sizes of 147, 63, 36, and 7 kb (54, 55), served as a size marker.

### Bacterial growth conditions.

E. coli strains were routinely cultured at 37°C on Luria-Bertani (LB) agar and in LB broth. For cell adhesion experiments, E. coli overnight cultures grown in LB broth were subcultured into Dulbecco’s modified Eagle medium (DMEM) with 0.45% glucose (Invitrogen).

### Cell adhesion assay.

The human colon carcinoma-derived cell line T84 was maintained in DMEM with nutrient mixture F-12 (DMEM–F-12) containing 2 mM l-glutamine (ATCC) supplemented with 10% fetal bovine serum (FBS). For the adhesion assay, cells were grown to 80% confluence in 24-well tissue culture plates (10^5^ cells/well), washed in DMEM (Invitrogen), and incubated with individual E. coli strains at a multiplicity of infection (MOI) of approximately 10 in DMEM for 1 h at 37°C. Quantification of cell-associated bacteria was performed by removing nonadhering bacteria, followed by washing and cell lysis. Serial dilutions of lysed cells with bacteria were plated and the number of CFU determined. Percent adherence was calculated by dividing the final number of CFU/ml by the initial number of CFU/ml for each replicate. Strain differences were calculated using a one-way analysis of variance (randomized design). *Post hoc* comparisons were performed using Tukey’s test. Both statistical tests were calculated with R v3.4.1.

### Data availability.

The accession numbers for the Illumina sequences generated from the 134 E. coli ST131 isolates presented in this study are available in the European Nucleotide Archive (ENA; https://www.ebi.ac.uk/ena) under accession number PRJEB27194. Sequences can also be located in the ENA using the following study summary: “Emergence of enteroaggregative Escherichia coli within the ST131 lineage as a cause of extraintestinal infections.”

The sequence of the pAA-ST131 plasmid has been deposited in GenBank under accession number KY706108.

## References

[1] Rogers BA, Sidjabat HE, Paterson DL 2011 *Escherichia coli* O25b-ST131: a pandemic, multiresistant, community-associated strain. J Antimicrob Chemother 66:1–14. doi:10.1093/jac/dkq415. 21081548

[2] Johnson JR, Johnston B, Clabots C, Kuskowski MA, Castanheira M 2010 *Escherichia coli* sequence type ST131 as the major cause of serious multidrug-resistant *E. coli* infections in the United States. Clin Infect Dis 51:286–294. doi:10.1086/653932. 20572763

[3] Banerjee R, Johnson JR 2014 A new clone sweeps clean: the enigmatic emergence of *Escherichia coli* sequence type 131. Antimicrob Agents Chemother 58:4997–5004. doi:10.1128/AAC.02824-14. 24867985 PMC4135879

[4] Stoesser N, Sheppard AE, Pankhurst L, De Maio N, Moore CE, Sebra R, Turner P, Anson LW, Kasarskis A, Batty EM, Kos V, Wilson DJ, Phetsouvanh R, Wyllie D, Sokurenko E, Manges AR, Johnson TJ, Price LB, Peto TE, Johnson JR, Didelot X, Walker AS, Crook DW, Modernizing Medical Microbiology Informatics Group. 2016 Evolutionary history of the global emergence of the *Escherichia coli* epidemic clone ST131. mBio 7:e02162. doi:10.1128/mBio.02162-15. 27006459 PMC4807372

[5] Ben Zakour NL, Alsheikh-Hussain AS, Ashcroft MM, Khanh Nhu NT, Roberts LW, Stanton-Cook M, Schembri MA, Beatson SA 2016 Sequential acquisition of virulence and fluoroquinolone resistance has shaped the evolution of *Escherichia coli* ST131. mBio 7:e00347-16. doi:10.1128/mBio.00347-16. 27118589 PMC4850260

[6] Price LB, Johnson JR, Aziz M, Clabots C, Johnston B, Tchesnokova V, Nordstrom L, Billig M, Chattopadhyay S, Stegger M, Andersen PS, Pearson T, Riddell K, Rogers P, Scholes D, Kahl B, Keim P, Sokurenko EV 2013 The epidemic of extended-spectrum-β-lactamase-producing *Escherichia coli* ST131 is driven by a single highly pathogenic subclone, H30-Rx. mBio 4:e00377-13. doi:10.1128/mBio.00377-13. 24345742 PMC3870262

[7] McNally A, Alhashash F, Collins M, Alqasim A, Paszckiewicz K, Weston V, Diggle M 2013 Genomic analysis of extra-intestinal pathogenic *Escherichia coli* urosepsis. Clin Microbiol Infect 19:E328–E334. doi:10.1111/1469-0691.12202. 23573792

[8] Matsumura Y, Johnson JR, Yamamoto M, Nagao M, Tanaka M, Takakura S, Ichiyama S, Kyoto-Shiga Clinical Microbiology Study Group. 2015 CTX-M-27- and CTX-M-14-producing, ciprofloxacin-resistant *Escherichia coli* of the H30 subclonal group within ST131 drive a Japanese regional ESBL epidemic. J Antimicrob Chemother 70:1639–1649. doi:10.1093/jac/dkv017. 25687644

[9] Liu CM, Stegger M, Aziz M, Johnson TJ, Waits K, Nordstrom L, Gauld L, Weaver B, Rolland D, Statham S, Horwinski J, Sariya S, Davis GS, Sokurenko E, Keim P, Johnson JR, Price LB 2018 *Escherichia coli* ST131-H22 as a foodborne uropathogen. mBio 9:e00470-18. doi:10.1128/mBio.00470-18. 30154256 PMC6113624

[10] Abe CM, Salvador FA, Falsetti IN, Vieira MA, Blanco J, Blanco JE, Blanco M, Machado AM, Elias WP, Hernandes RT, Gomes TA 2008 Uropathogenic *Escherichia coli* (UPEC) strains may carry virulence properties of diarrhoeagenic *E. coli*. FEMS Immunol Med Microbiol 52:397–406. doi:10.1111/j.1574-695X.2008.00388.x. 18336383

[11] Blanco J, Mora A, Mamani R, López C, Blanco M, Dahbi G, Herrera A, Marzoa J, Fernández V, de la Cruz F, Martínez-Martínez L, Alonso MP, Nicolas-Chanoine MH, Johnson JR, Johnston B, López-Cerero L, Pascual A, Rodríguez-Baño J, Spanish Group for Nosocomial Infections. 2013 Four main virotypes among extended-spectrum-β-lactamase-producing isolates of *Escherichia coli* O25b:H4-B2-ST131: bacterial, epidemiological, and clinical characteristics. J Clin Microbiol 51:3358–3367. doi:10.1128/JCM.01555-13. 23926164 PMC3811668

[12] Olesen B, Frimodt-Møller J, Leihof RF, Struve C, Johnston B, Hansen DS, Scheutz F, Krogfelt KA, Kuskowski MA, Clabots C, Johnson JR 2014 Temporal trends in antimicrobial resistance and virulence-associated traits within the *Escherichia coli* sequence type 131 clonal group and its H30 and H30-Rx subclones, 1968 to 2012. Antimicrob Agents Chemother 58:6886–6895. doi:10.1128/AAC.03679-14. 25199783 PMC4249411

[13] Hebbelstrup Jensen B, Olsen KE, Struve C, Krogfelt KA, Petersen AM 2014 Epidemiology and clinical manifestations of enteroaggregative *Escherichia coli*. Clin Microbiol Rev 27:614–630. doi:10.1128/CMR.00112-13. 24982324 PMC4135892

[14] Rasko DA, Webster DR, Sahl JW, Bashir A, Boisen N, Scheutz F, Paxinos EE, Sebra R, Chin CS, Iliopoulos D, Klammer A, Peluso P, Lee L, Kislyuk AO, Bullard J, Kasarskis A, Wang S, Eid J, Rank D, Redman JC, Steyert SR, Frimodt-Møller J, Struve C, Petersen AM, Krogfelt KA, Nataro JP, Schadt EE, Waldor MK 2011 Origins of the *E. coli* strain causing an outbreak of hemolytic-uremic syndrome in Germany. N Engl J Med 365:709–717. doi:10.1056/NEJMoa1106920. 21793740 PMC3168948

[15] Imuta N, Ooka T, Seto K, Kawahara R, Koriyama T, Kojyo T, Iguchi A, Tokuda K, Kawamura H, Yoshiie K, Ogura Y, Hayashi T, Nishi J 2016 Phylogenetic analysis of enteroaggregative *Escherichia coli* (EAEC) isolates from Japan reveals emergence of CTX-M-14-producing EAEC O25:H4 clones related to sequence type 131. J Clin Microbiol 54:2128–2134. doi:10.1128/JCM.00711-16. 27252465 PMC4963495

[16] Guiral E, Mendez-Arancibia E, Soto SM, Salvador P, Fabrega A, Gascon J, Vila J 2011 CTX-M-15-producing enteroaggregative *Escherichia coli* as cause of travelers’ diarrhea. Emerg Infect Dis 17:1950–1953. doi:10.3201/eid1710.110022. 22000380 PMC3310664

[17] Amaya E, Reyes D, Vilchez S, Paniagua M, Möllby R, Nord CE, Weintraub A 2011 Antibiotic resistance patterns of intestinal *Escherichia coli* isolates from Nicaraguan children. J Med Microbiol 60:216–222. doi:10.1099/jmm.0.020842-0. 20965916

[18] Zhang R, Gu DX, Huang YL, Chan EW, Chen GX, Chen S 2016 Comparative genetic characterization of Enteroaggregative *Escherichia coli* strains recovered from clinical and non-clinical settings. Sci Rep 6:24321. doi:10.1038/srep24321. 27062991 PMC4827025

[19] Santiago AE, Ruiz-Perez F, Jo NY, Vijayakumar V, Gong MQ, Nataro JP 2014 A large family of antivirulence regulators modulates the effects of transcriptional activators in Gram-negative pathogenic bacteria. PLoS Pathog 10:e1004153. doi:10.1371/journal.ppat.1004153. 24875828 PMC4038620

[20] Nataro JP 2005 Enteroaggregative Escherichia coli pathogenesis. Curr Opin Gastroenterol 21:4–8. 15687877

[21] Boisen N, Scheutz F, Rasko DA, Redman JC, Persson S, Simon J, Kotloff KL, Levine MM, Sow S, Tamboura B, Toure A, Malle D, Panchalingam S, Krogfelt KA, Nataro JP 2012 Genomic characterization of enteroaggregative *Escherichia coli* from children in Mali. J Infect Dis 205:431–444. doi:10.1093/infdis/jir757. 22184729 PMC3256949

[22] Jønsson R, Struve C, Boisen N, Mateiu RV, Santiago AE, Jenssen H, Nataro JP, Krogfelt KA 2015 Novel aggregative adherence fimbria variant of enteroaggregative *Escherichia coli*. Infect Immun 83:1396–1405. doi:10.1128/IAI.02820-14. 25624357 PMC4363450

[23] Morin N, Santiago AE, Ernst RK, Guillot SJ, Nataro JP 2013 Characterization of the AggR regulon in enteroaggregative *Escherichia coli*. Infect Immun 81:122–132. doi:10.1128/IAI.00676-12. 23090962 PMC3536136

[24] Roer L, Hansen F, Thomsen MCF, Knudsen JD, Hansen DS, Wang M, Samulioniené J, Justesen US, Røder BL, Schumacher H, Østergaard C, Andersen LP, Dzajic E, Søndergaard TS, Stegger M, Hammerum AM, Hasman H 2017 WGS-based surveillance of third-generation cephalosporin-resistant *Escherichia coli* from bloodstream infections in Denmark. J Antimicrob Chemother 72:1922–1929. doi:10.1093/jac/dkx092. 28369408

[25] Johnson TJ, Nolan LK 2009 Pathogenomics of the virulence plasmids of *Escherichia coli*. Microbiol Mol Biol Rev 73:750–774. doi:10.1128/MMBR.00015-09. 19946140 PMC2786578

[26] Johnson JR, Murray AC, Gajewski A, Sullivan M, Snippes P, Kuskowski MA, Smith KE 2003 Isolation and molecular characterization of nalidixic acid-resistant extraintestinal pathogenic *Escherichia coli* from retail chicken products. Antimicrob Agents Chemother 47:2161–2168. doi:10.1128/AAC.47.7.2161-2168.2003. 12821463 PMC161843

[27] Spurbeck RR, Dinh PC, Walk ST, Stapleton AE, Hooton TM, Nolan LK, Kim KS, Johnson JR, Mobley HL 2012 *Escherichia coli* isolates that carry *vat*, *fyuA*, *chuA*, and *yfcV* efficiently colonize the urinary tract. Infect Immun 80:4115–4122. doi:10.1128/IAI.00752-12. 22966046 PMC3497434

[28] Coelho A, Mora A, Mamani R, López C, González-López JJ, Larrosa MN, Quintero-Zarate JN, Dahbi G, Herrera A, Blanco JE, Blanco M, Alonso MP, Prats G, Blanco J 2011 Spread of *Escherichia coli* O25b:H4-B2-ST131 producing CTX-M-15 and SHV-12 with high virulence gene content in Barcelona (Spain). J Antimicrob Chemother 66:517–526. doi:10.1093/jac/dkq491. 21177675

[29] Lanza VF, de Toro M, Garcillán-Barcia MP, Mora A, Blanco J, Coque TM, de la Cruz F 2014 Plasmid flux in *Escherichia coli* ST131 sublineages, analyzed by plasmid constellation network (PLACNET), a new method for plasmid reconstruction from whole genome sequences. PLoS Genet 10:e1004766. doi:10.1371/journal.pgen.1004766. 25522143 PMC4270462

[30] Okhuysen PC, Dupont HL 2010 Enteroaggregative *Escherichia coli* (EAEC): a cause of acute and persistent diarrhea of worldwide importance. J Infect Dis 202:503–505. doi:10.1086/654895. 20594108

[31] Itoh Y, Nagano I, Kunishima M, Ezaki T 1997 Laboratory investigation of enteroaggregative *Escherichia coli* O untypeable:H10 associated with a massive outbreak of gastrointestinal illness. J Clin Microbiol 35:2546–2550. doi:10.1128/JCM.35.10.2546-2550.1997. 9316905 PMC230008

[32] Dallman TJ, Chattaway MA, Cowley LA, Doumith M, Tewolde R, Wooldridge DJ, Underwood A, Ready D, Wain J, Foster K, Grant KA, Jenkins C 2014 An investigation of the diversity of strains of enteroaggregative *Escherichia coli* isolated from cases associated with a large multi-pathogen foodborne outbreak in the UK. PLoS One 9:e98103. doi:10.1371/journal.pone.0098103. 24844597 PMC4028294

[33] Park HK, Jung YJ, Chae HC, Shin YJ, Woo SY, Park HS, Lee SJ 2009 Comparison of *Escherichia coli* uropathogenic genes (*kps*, *usp* and *ireA*) and enteroaggregative genes (*aggR* and *aap*) via multiplex polymerase chain reaction from suprapubic urine specimens of young children with fever. Scand J Urol Nephrol 43:51–57. doi:10.1080/00365590802299338. 18759167

[34] Olesen B, Scheutz F, Andersen RL, Menard M, Boisen N, Johnston B, Hansen DS, Krogfelt KA, Nataro JP, Johnson JR 2012 Enteroaggregative *Escherichia coli* O78:H10–the cause of an outbreak of urinary tract infection. J Clin Microbiol 50:3703–3711. doi:10.1128/JCM.01909-12. 22972830 PMC3486220

[35] Boll EJ, Struve C, Boisen N, Olesen B, Stahlhut SG, Krogfelt KA 2013 Role of enteroaggregative *Escherichia coli* virulence factors in uropathogenesis. Infect Immun 81:1164–1171. doi:10.1128/IAI.01376-12. 23357383 PMC3639612

[36] Okeke IN, Wallace-Gadsden F, Simons HR, Matthews N, Labar AS, Hwang J, Wain J 2010 Multi-locus sequence typing of enteroaggregative *Escherichia coli* isolates from Nigerian children uncovers multiple lineages. PLoS One 5:e14093. doi:10.1371/journal.pone.0014093. 21124856 PMC2990770

[37] Chattaway MA, Jenkins C, Ciesielczuk H, Day M, DoNascimento V, Day M, Rodríguez I, van Essen-Zandbergen A, Schink A-K, Wu G, Threlfall J, Woodward MJ, Coldham N, Kadlec K, Schwarz S, Dierikx C, Guerra B, Helmuth R, Mevius D, Woodford N, Wain J 2014 Evidence of evolving extraintestinal enteroaggregative *Escherichia coli* ST38 clone. Emerg Infect Dis 20:1935–1937. doi:10.3201/eid2011.131845. 25340736 PMC4214294

[38] Zhang J, Zheng B, Zhao L, Wei Z, Ji J, Li L, Xiao Y 2014 Nationwide high prevalence of CTX-M and an increase of CTX-M-55 in *Escherichia coli* isolated from patients with community-onset infections in Chinese county hospitals. BMC Infect Dis 14:659. doi:10.1186/s12879-014-0659-0. 25466590 PMC4265337

[39] Xia S, Fan X, Huang Z, Xia L, Xiao M, Chen R, Xu Y, Zhuo C 2014 Dominance of CTX-M-type extended-spectrum β-lactamase (ESBL)-producing *Escherichia coli* isolated from patients with community-onset and hospital-onset infection in China. PLoS One 9:e100707. doi:10.1371/journal.pone.0100707. 24983621 PMC4077569

[40] Tong P, Sun Y, Ji X, Du X, Guo X, Liu J, Zhu L, Zhou B, Zhou W, Liu G, Feng S 2015 Characterization of antimicrobial resistance and extended-spectrum β-lactamase genes in *Escherichia coli* isolated from chickens. Foodborne Pathog Dis 12:345–352. doi:10.1089/fpd.2014.1857. 25785885

[41] Mitsumori K, Terai A, Yamamoto S, Yoshida O 1997 Virulence characteristics and DNA fingerprints of *Escherichia* coli isolated from women with acute uncomplicated pyelonephritis. J Urol 158:2329–2332. doi:10.1016/s0022-5347(01)68244-2. 9366385

[42] Frank C, Werber D, Cramer JP, Askar M, Faber M, An der Heiden M, Bernard H, Fruth A, Prager R, Spode A, Wadl M, Zoufaly A, Jordan S, Kemper MJ, Follin P, Müller L, King LA, Rosner B, Buchholz U, Stark K, Krause G, HUS Investigation Team. 2011 Epidemic profile of Shiga-toxin-producing *Escherichia coli* O104:H4 outbreak in Germany. N Engl J Med 365:1771–1780. doi:10.1056/NEJMoa1106483. 21696328

[43] Thomsen MC, Ahrenfeldt J, Cisneros JL, Jurtz V, Larsen MV, Hasman H, Aarestrup FM, Lund O 2016 A bacterial analysis platform: an integrated system for analysing bacterial whole genome sequencing data for clinical diagnostics and surveillance. PLoS One 11:e0157718. doi:10.1371/journal.pone.0157718. 27327771 PMC4915688

[44] Aziz RK, Bartels D, Best AA, DeJongh M, Disz T, Edwards RA, Formsma K, Gerdes S, Glass EM, Kubal M, Meyer F, Olsen GJ, Olson R, Osterman AL, Overbeek RA, McNeil LK, Paarmann D, Paczian T, Parrello B, Pusch GD, Reich C, Stevens R, Vassieva O, Vonstein V, Wilke A, Zagnitko O 2008 The RAST Server: rapid annotations using subsystems technology. BMC Genomics 9:75. doi:10.1186/1471-2164-9-75. 18261238 PMC2265698

[45] Alikhan NF, Petty NK, Ben Zakour NL, Beatson SA 2011 BLAST Ring Image Generator (BRIG): simple prokaryote genome comparisons. BMC Genomics 12:402. doi:10.1186/1471-2164-12-402. 21824423 PMC3163573

[46] Sahl JW, Lemmer D, Travis J, Schupp JM, Gillece JD, Aziz M, Driebe EM, Drees KP, Hicks ND, Williamson CHD, Hepp CM, Smith DE, Roe C, Engelthaler DM, Wagner DM, Keim P 2016 NASP: an accurate, rapid method for the identification of SNPs in WGS datasets that supports flexible input and output formats. Microb Genom 2:e000074. doi:10.1099/mgen.0.000074. 28348869 PMC5320593

[47] Delcher AL, Phillippy A, Carlton J, Salzberg SL 2002 Fast algorithms for large-scale genome alignment and comparison. Nucleic Acids Res 30:2478–2483. doi:10.1093/nar/30.11.2478. 12034836 PMC117189

[48] Li H, Durbin R 2009 Fast and accurate short read alignment with Burrows-Wheeler transform. Bioinformatics 25:1754–1760. doi:10.1093/bioinformatics/btp324. 19451168 PMC2705234

[49] McKenna A, Hanna M, Banks E, Sivachenko A, Cibulskis K, Kernytsky A, Garimella K, Altshuler D, Gabriel S, Daly M, DePristo MA 2010 The Genome Analysis Toolkit: a MapReduce framework for analyzing next-generation DNA sequencing data. Genome Res 20:1297–1303. doi:10.1101/gr.107524.110. 20644199 PMC2928508

[50] Croucher NJ, Page AJ, Connor TR, Delaney AJ, Keane JA, Bentley SD, Parkhill J, Harris SR 2015 Rapid phylogenetic analysis of large samples of recombinant bacterial whole genome sequences using Gubbins. Nucleic Acids Res 43:e15. doi:10.1093/nar/gku1196. 25414349 PMC4330336

[51] Stamatakis A 2014 RAxML version 8: a tool for phylogenetic analysis and post-analysis of large phylogenies. Bioinformatics 30:1312–1313. doi:10.1093/bioinformatics/btu033. 24451623 PMC3998144

[52] Lefort V, Longueville JE, Gascuel O 2017 SMS: smart model selection in PhyML. Mol Biol Evol 34:2422–2424. doi:10.1093/molbev/msx149. 28472384 PMC5850602

[53] Kado CI, Liu ST 1981 Rapid procedure for detection and isolation of large and small plasmids. J Bacteriol 145:1365–1373. doi:10.1128/JB.145.3.1365-1373.1981. 7009583 PMC217141

[54] Threlfall EJ, Rowe B, Ferguson JL, Ward LR 1986 Characterization of plasmids conferring resistance to gentamicin and apramycin in strains of *Salmonella typhimurium* phage type 204c isolated in Britain. J Hyg (Lond) 97:419–426. doi:10.1017/s0022172400063609. 3540111 PMC2082898

[55] Schjørring S, Struve C, Krogfelt KA 2008 Transfer of antimicrobial resistance plasmids from *Klebsiella pneumoniae* to *Escherichia coli* in the mouse intestine. J Antimicrob Chemother 62:1086–1093. doi:10.1093/jac/dkn323. 18703526 PMC2566516

