# Supplementary figures and images for: Emergence of Enteroaggregative Escherichia coli within the ST131 Lineage as a Cause of Extraintestinal Infections
Source: mBio. 2020 May 19;11(3):e00353-20. doi: 10.1128/mBio.00353-20 (PMC7240153; doi:10.1128/mBio.00353-20)

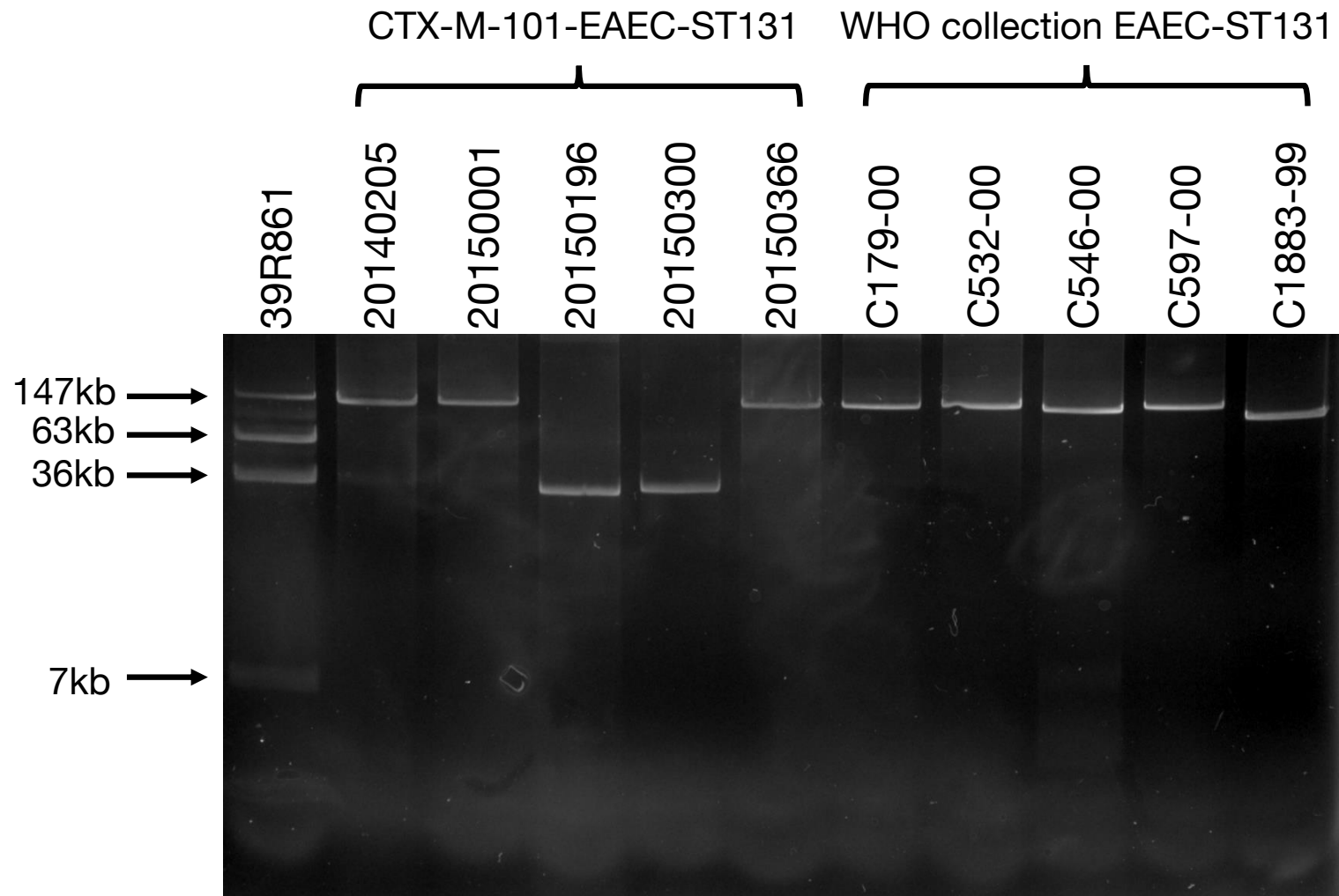

Supplement: FIG S2 [file mBio.00353-20-sf002.pdf]

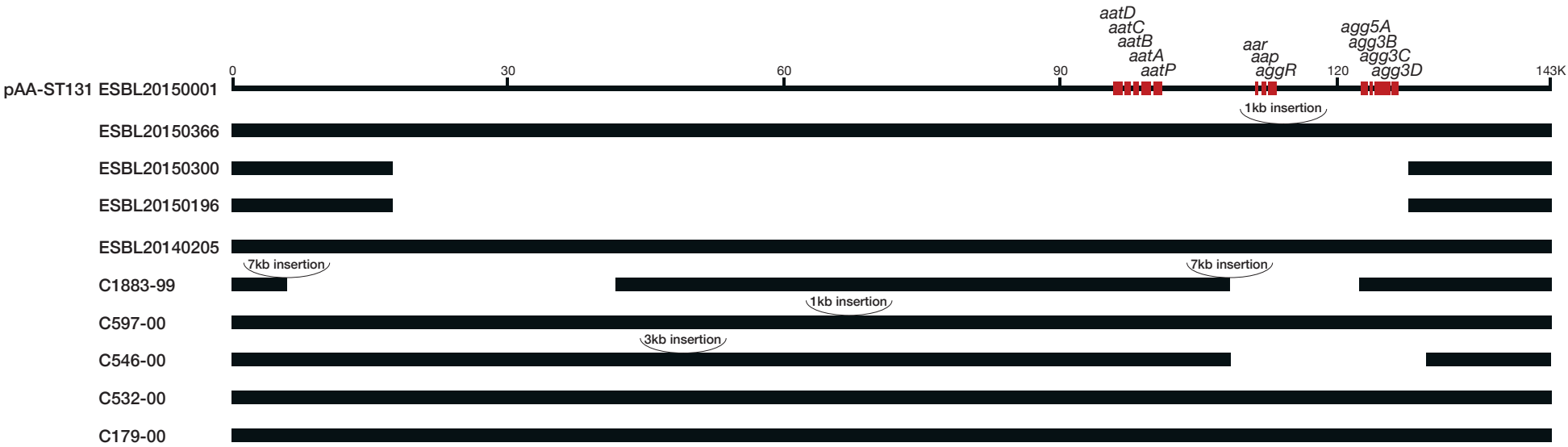

Supplement: FIG S3 [file mBio.00353-20-sf003.pdf]

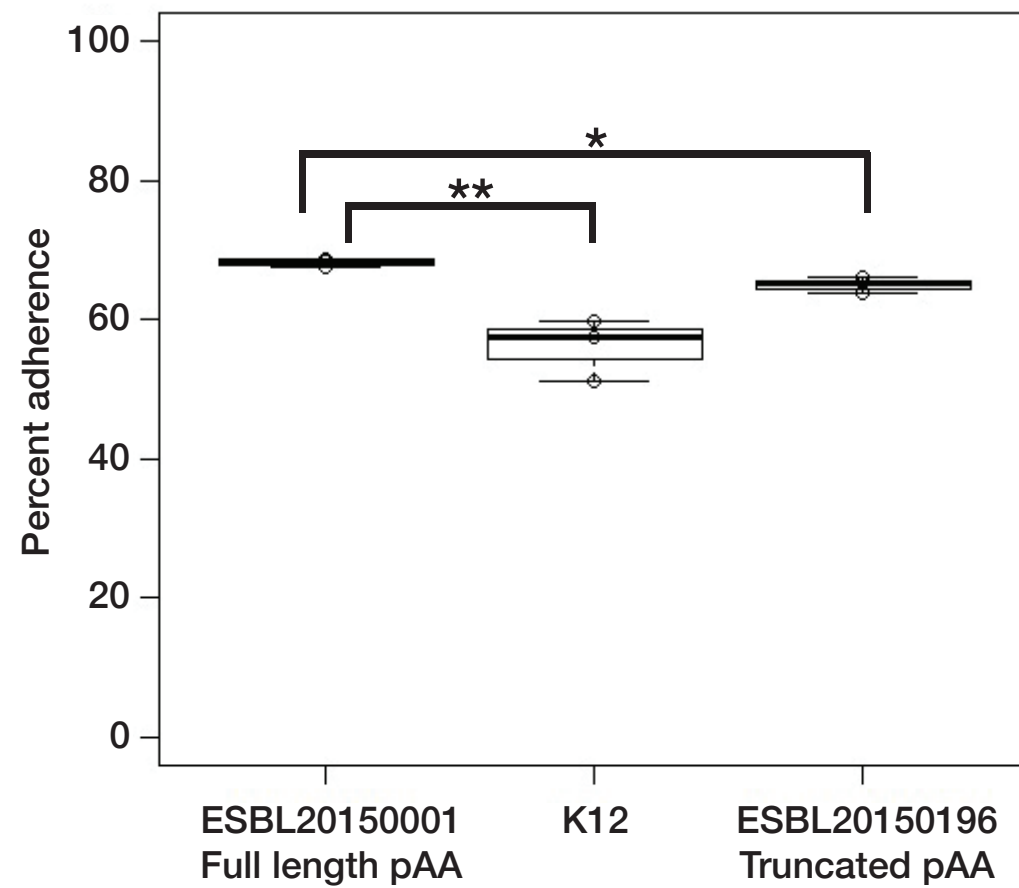

Supplement: FIG S5 [file mBio.00353-20-sf005.pdf]
