# Supplementary material for: Emergence of Enteroaggregative Escherichia coli within the ST131 Lineage as a Cause of Extraintestinal Infections
Source: mBio. 2020 May 19;11(3):e00353-20. doi: 10.1128/mBio.00353-20 (PMC7240153; doi:10.1128/mBio.00353-20)
Supplement: FIG S4 [file mBio.00353-20-sf004.pdf]

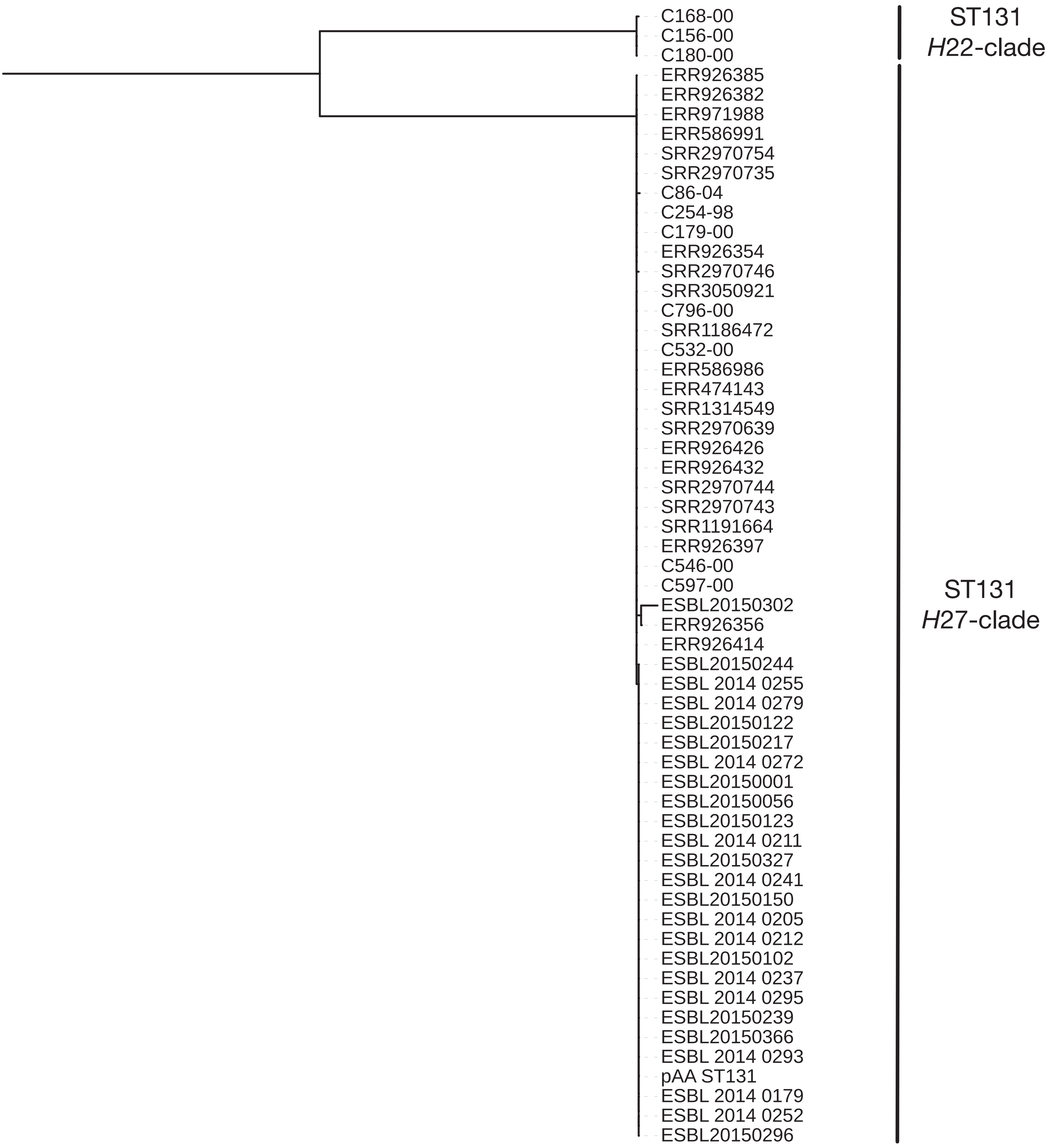
C168-00
C156-00
C180-00
ERR926385
ERR926382
ERR971988
ERR586991
SRR2970754
SRR2970735
C86-04
C254-98
C179-00
ERR926354
SRR2970746
SRR3050921
C796-00
SRR1186472
C532-00
ERR586986
ERR474143
SRR1314549
SRR2970639
ERR926426
ERR926432
SRR2970744
SRR2970743
SRR1191664
ERR926397
C546-00
C597-00
ESBL20150302
ERR926356
ERR926414
ESBL20150244
ESBL 2014 0255
ESBL 2014 0279
ESBL20150122
ESBL20150217
ESBL 2014 0272
ESBL20150001
ESBL20150056
ESBL20150123
ESBL 2014 0211
ESBL20150327
ESBL 2014 0241
ESBL20150150
ESBL 2014 0205
ESBL 2014 0212
ESBL20150102
ESBL 2014 0237
ESBL 2014 0295
ESBL20150239
ESBL20150366
ESBL 2014 0293
pAA ST131
ESBL 2014 0179
ESBL 2014 0252
ESBL20150296
ST131
*H*22-clade
ST131
*H*27-clade
