# Supplementary material for: Emergence of Enteroaggregative Escherichia coli within the ST131 Lineage as a Cause of Extraintestinal Infections
Source: mBio. 2020 May 19;11(3):e00353-20. doi: 10.1128/mBio.00353-20 (PMC7240153; doi:10.1128/mBio.00353-20)
Supplement: FIG S1 [file mBio.00353-20-sf001.pdf]

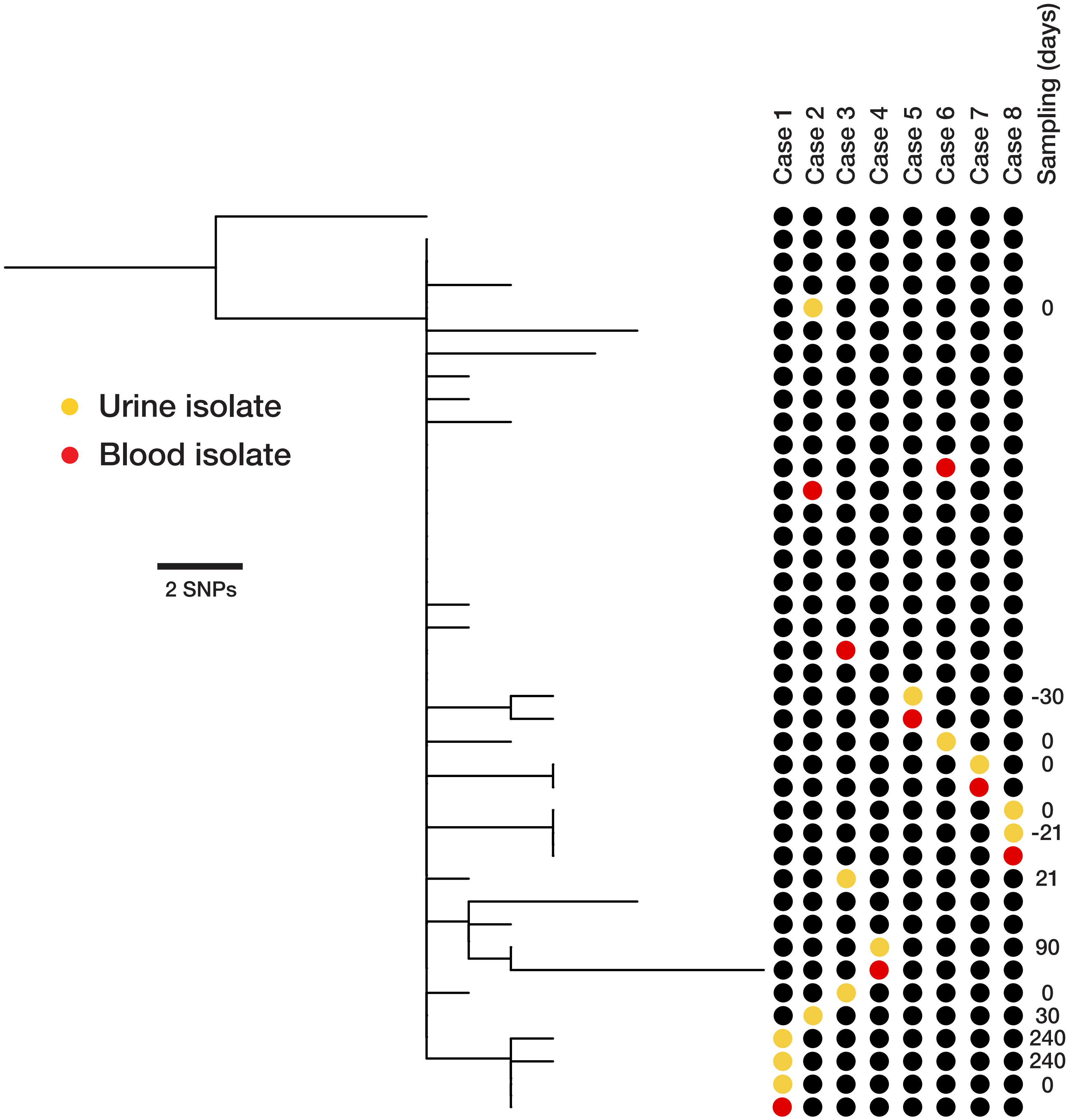

Urine isolate
Blood isolate
2 SNPs
Case 1
Case 2
Case 3
Case 4
Case 5
Case 6
Case 7
Case 8
Sampling (days)
0
-30
0
0
0
-21
21
90
0
30
240
240
0
